# The Olmsted Bill

**Published:** 1898-09

**Authors:** 


### THE OLMSTED BILL.

In our News and Notes of this issue will be found a clipping from the *Medico-Surgical Bulletin*, in which its editor congratulates Dr. Olmsted, of this city, on having been paid his fee of $500.00 by the Board of Health for services rendered the city in treating a case of yellow fever. Our readers, no doubt, will remember the circumstances, and the fact that the Board of Health refused to pay the bill of $500.00 rendered by Dr. Olmsted on the ground that the fee was too large. Nearly every medical journal in the country passed comments on this action of the Board of Health, and without exception they criticized its action. Soon after this action of the board its personnel was changed under the varying fortunes of resignations and politics, so that the matter of the "Olmsted Bill" was again brought up, and this time unanimously passed, recommending to the city its payment. We thought this would end the matter, but now comes the startling fact that the city refuses to pay Dr. Olmsted, on the grounds that they cannot take the money out of the appropriation to the Sanitary Department, and that there is no available money from which such a payment could be made. It looks like a plain case of the city trying "to do" Dr. Olmsted, just as physicians are "done" frequently by individual members of the community. It matters not from what source the city gets the money, the fact remains simply this: that the city is obligated for this bill, and Dr. Olmsted ought to be paid immediately. If ever there was a legitimate payment from the city's funds, then this is the case; for if this should be the only black mark registered against the city for misappropriated funds, then our city fathers would wear white wings from henceforth and forever. We want to see Dr. Olmsted get what he deserves, and he deserves every cent of the five hundred dollars.

